# Investigating CAR-T Treatment Access for Multiple Myeloma Patients Using Real-World Evidence

**DOI:** 10.3390/cancers18040669

**Published:** 2026-02-18

**Authors:** Jaysón Davidson, Anupama Kumar, Ayan Patel, Irene Y. Chen, Atul J. Butte, Travis Zack

**Affiliations:** 1 Bakar Computational Health Sciences Institute, University of California San Francisco, San Francisco, CA 94158, USA; 2 Center for Data-Driven Insights and Innovation, University of California Health, Oakland, CA 94607, USA; 3 Department of Medicine, Division of Hematology and Oncology, University of California San Francisco, San Francisco, CA 94143, USA; 4 Computational Precision Health, University of California San Francisco, San Francisco, CA 94158, USA; 5 University of California Berkeley, Berkeley, CA 94117, USA

**Keywords:** multiple myeloma, CAR-T eligibility, healthcare disparities, CAR-T therapy

## Abstract

**Importance**: Multiple myeloma (MM) is the second most common hematologic malignancy in the U.S., with a higher incidence among Black patients than White patients. Chimeric antigen receptor T-cell (CAR-T) therapies show clinical promise, but their limited availability raises concerns about access. **Objective**: To examine associations between disease characteristics, treatment location, and patient demographics with receipt of CAR-T therapy among patients with MM. **Design**: Retrospective cohort study using electronic health record data from the University of California Health Data Warehouse (UCHDW) between January 2021 and January 2025. **Setting**: Six academic health centers and twelve affiliated hospitals within the UCHDW. **Participants**: A population-based cohort of 12,360 adult patients diagnosed with MM and treated at a University of California facility offering CAR-T administration. Analyses were conducted from February 2025 to March 2025. **Exposures**: Receipt of multiple cancer therapies following MM diagnosis. **Main Outcomes and Measures**: Logistic regression was used to estimate odds ratios (ORs) and 95% confidence intervals (CIs) for associations between disease characteristics, treatment locations, and patient demographics with receipt of CAR-T therapy. A zero-shot GPT-4 inference model was applied to UCSF clinical notes to assess whether CAR-T therapy was discussed, determine documented eligibility, and classify rationale for eligibility determinations. **Results**: Among 12,360 patients with MM (mean age, 68.5 years; 51.6% male), 320 (2.6%) received CAR-T therapy. Disease characteristics at diagnosis, measured by the International Staging System (ISS), was distributed as follows: Stage I (65.3%), Stage II (24.4%), Stage III (2.8%), and Unknown (7.5%). Patients treated at UC-1 (49.3%), and UC-2 (50.0%) were more frequently diagnosed with ISS Stage II, whereas patients treated at UC-3 (55.5%) were more frequently diagnosed with ISS Stage I. Our model showed that patients identifying as Black or African American had lower odds of receiving CAR-T therapy compared with White patients (OR, 0.33; [95% CI, 0.17–0.62]). Patients treated at UC-3 also had lower odds of receiving CAR-T therapy compared with UC-1 (OR, 0.42; [95% CI, 0.30–0.59]). Among 270 UCSF patients assessed for CAR-T eligibility using clinical notes, the proportion of patients deemed eligible without documented CAR-T discussions was highest among those identifying as Other Pacific Islander (50%), followed by Black or African American (4.2%), Asian (3.2%), and White patients (0.6%). **Conclusions and Relevance**: Within a large academic health system, receipt of CAR-T therapy varied by treatment location and patient-reported race. A subset of patients with documented eligibility lacked recorded discussions of CAR-T therapy, suggesting potential differences in referral, documentation, or care pathways influencing observed treatment patterns.

## Simple Summary

Multiple myeloma is a common hematologic cancer in the U.S., with Black patients affected more often than White patients, yet access to new treatments like CAR-T therapy remains limited. This study used real-world data from the University of California health system to examine how disease characteristics, treatment location, and patient demographics are associated with CAR-T therapy receipt. We found that both treatment location and patient-reported race were associated with differences in CAR-T receipt, even after accounting for disease severity and insurance coverage. Using a GPT-4 model to analyze clinical notes, we identified patients eligible for CAR-T therapy who had no documented discussions about it. These findings highlight potential gaps in method of care and provider communication that could limit access to advanced therapies. By uncovering these patterns, this research may help clinicians, health systems, and researchers develop strategies to ensure that patients have better access to receiving advanced treatments.

## 1. Introduction

Multiple myeloma (MM) is a hematologic malignancy characterized by the clonal proliferation of malignant plasma cells, which can result in end-organ damage, morbidity, and mortality (with a five-year overall survival of 58%) [1]. Black patients are diagnosed at twice the rate and, on average, 5–10 years earlier than White patients [2] and are more likely to present with anemia, hypercalcemia, and kidney dysfunction [3,4]. Despite this higher disease burden, Black patients are underrepresented in clinical trials evaluating novel therapies [5,6]; although real-world studies indicate that when given equal access to care, Black patients achieve similar or better survival than White patients [3,6,7,8]. Hispanic or Latino patients with MM also present at younger ages and experience longer intervals from diagnosis to therapy initiation, with lower utilization of essential therapies such as autologous stem cell transplant (ASCT) [9].

Standard-of-care front-line therapy consists of triplet or quadruplet induction therapy, followed by ASCT consolidation and maintenance for eligible patients, while transplant-ineligible patients receive modified ongoing systemic therapy [10,11,12,13,14]. Despite these advances, MM invariably relapses. Recently, chimeric antigen receptor T-cell (CAR-T) therapy, engineered to target the B-cell maturation antigen (BCMA) on plasma cells, has emerged as a breakthrough for relapsed/refractory MM [15,16]. Two FDA-approved BCMA CAR-T therapies, idecabtagene vicleucel (ide-cel) and ciltacabtagene autoleucel (cilta-cel), have demonstrated unprecedented outcomes, including up to 98% overall response rate (ORR) and nearly 36-month progression-free survival (PFS) with cilta-cel [17], and offer the appeal of a treatment-free interval (TFI). However, CAR-T therapy is only available at a limited number of academic centers with expertise in CAR-T management and related toxicities. Patients without access to specialized centers, the ability to relocate, or adequate social support for caregiving may face significant barriers. These barriers may disproportionately affect Black and Hispanic or Latino patients, but the extent to which they influence access to CAR-T therapy remains unclear.

We hypothesize that patient race and treatment location are associated with CAR-T therapy receipt among MM patients, considering both disease risk and baseline demographics. To test this hypothesis, we analyzed associations between CAR-T receipt, disease severity, and patient demographics across University of California (UC) Health system locations. Our study leveraged a clinical data warehouse covering over nine million individuals across three UC academic medical centers offering CAR-T therapy: UCLA, UCSD, and UCSF.

## 2. Methods

### 2.1. Data Supporting Study: University of California Health Data Warehouse (UCHDW)

Data were drawn from the University of California Health system, which includes 20 health professional schools (6 medical schools), 5 academic health centers (UC San Francisco, UC Los Angeles, UC Davis, UC Irvine, and UC San Diego), and 12 hospitals. It has built a secure central data warehouse (UCHDW) for operational improvement and promotion of quality patient care, enabling the next generation of clinical research [18]. The repository currently holds data securely on over 9 million patients seen since 2012. EHR data is extracted from each site and transformed into the vendor-neutral Observational Medical Outcomes Partnership (OMOP) common data model [19]. De-identification of the data has been completed to enable clinical research projects, under guidance from UC campus institutional review boards (IRBs), privacy, and compliance. Research using UCHDW data is considered non-human-subject research under IRB guidance.

### 2.2. Study Population

**Figure 1:**
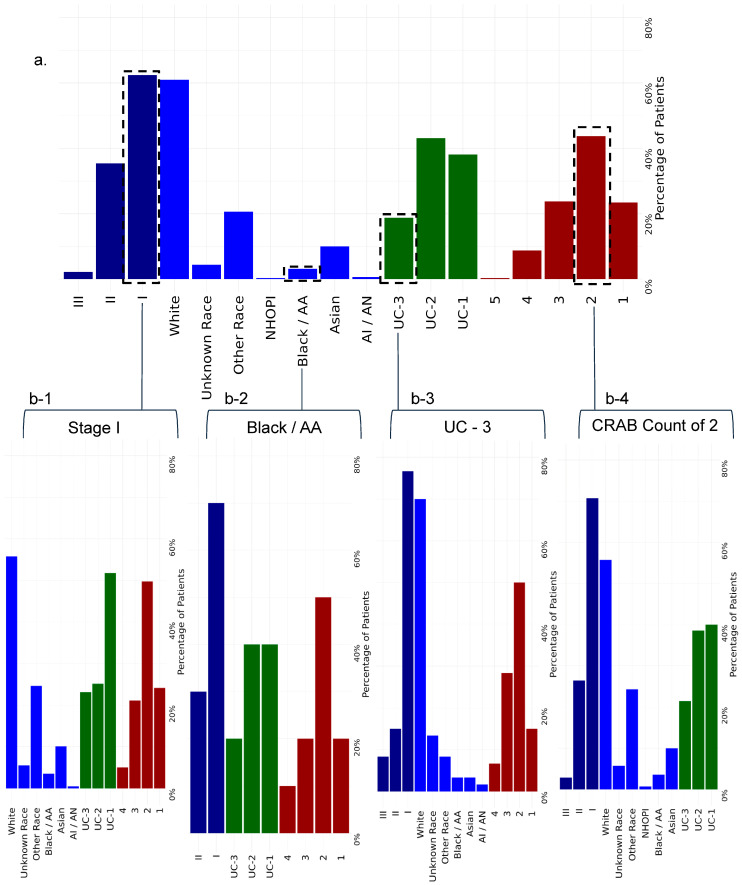

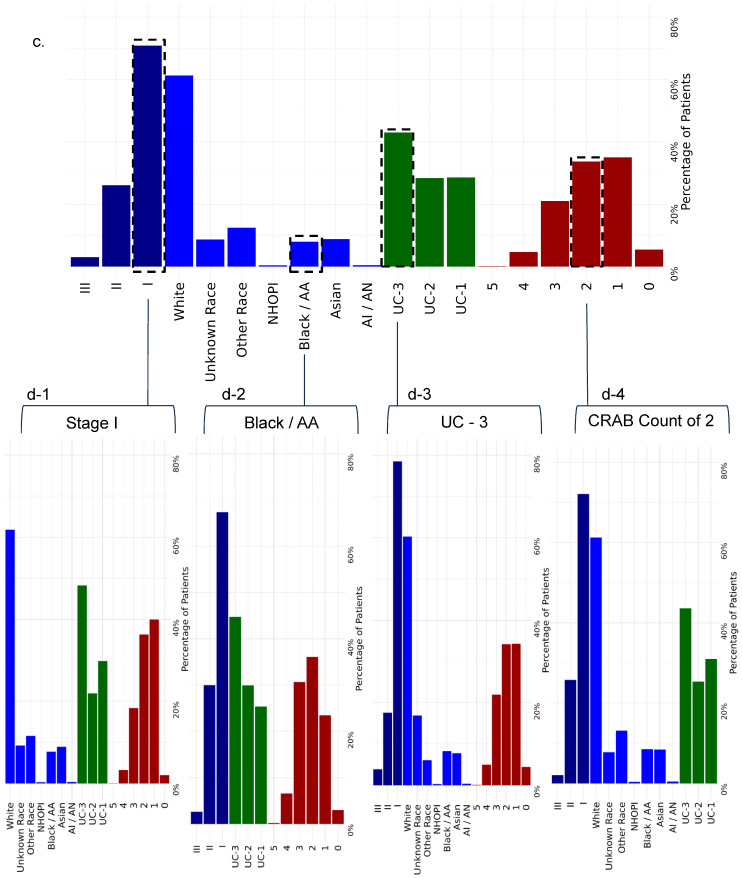
(**a**) Distribution of demographics for patients who received CAR-T therapy. (**b-1**) Patients diagnosed with ISS Stage I. (**b-2**) Patients who identified as Black or African American. (**b-3**) Patients who were treated at UC-3. (**b-4**) Patients with 2 CRAB features. (**c**,**d-1**–**d-4**) Patients who did not receive CART. (**c**) Distribution of demographics for patients who did not receive CAR-T therapy. (**d-1**) Patients diagnosed with ISS Stage I. (**d-2**) Patients who identified as Black or African American. (**d-3**) Patients who were treated at UC-3. (**d-4**) Patients with 2 CRAB features.

We identified patients with MM ICD-10 code (C90.0) who had documentation for multiple cancer therapies and procedures from EHRs spanning from 2012 to February 2025 (Supplement S1). To focus on potential CAR-T recipients, we used the following inclusion/exclusion criteria to create a final study cohort of 12,360 patients (Figure 1). If patients diagnosed with MM did not have primary insurance information, they were excluded from our study to include only patients with insurance. Patients without an Area Deprivation Index (ADI) score were excluded. We further excluded patients without a visit on or after 1 January 2021, because eligibility for non-investigational CAR-T currently requires at least one previous line of therapy. We considered all therapies patients received after MM diagnosis, with at least one year of treatment. Patients who did not have more than one cancer treatment were excluded from our study. Patients who were treated at a UC facility offering CAR-T administration (UCSF, UCLA, or UCSD) after January 2021 were included in our study. Finally, all patients under 18 years of age at the time of diagnosis were excluded from the cohort. The study adhered to the Strengthening the Reporting of Observational Studies in Epidemiology (STROBE) reporting guidelines [20].

### 2.3. Area Deprivation Index (ADI)

We utilized ADI as a proxy for patients’ socioeconomic status [21]. ADI does not account for specific individual or family characteristics but is a general estimate of SDOH based on home location census tract characteristics by integrating data from 17 different variables sourced from the U.S. census, with factors related to poverty, housing, employment, and education. ADI as a metric has been used in previous observational studies [22,23,24,25,26] to understand its SDOH role on health outcomes in other disease states. Higher percentile scores indicate greater neighborhood disadvantage [21,27,28,29].

### 2.4. Study Variables

CAR-T receipt was identified through UCHDW data. Covariates included ADI, age (in years), patient-reported gender (Male, Female), presence of primary insurance coverage, patient-reported race, ethnicity, UC location, and International Staging System (ISS) disease stage at prognosis. To identify cancer procedures and therapies for patients, we categorized all treatments (surgery, chemotherapy, radiation, ASCT, monoclonal antibodies, corticosteroids, alkylators, bispecific antibodies, CAR-T, immunomodulatory agents, nuclear export inhibitors, or proteasome inhibitors) to ensure that we correctly accounted for cancer therapeutic classes before CAR-T treatments. The International Staging System (ISS) stage was calculated using serum albumin and serum β2 microglobulin (Sβ2M) levels [30]. Patients were categorized as ISS Stage I, II, III, or None as defined by patients whose ISS could not be calculated. Associations observed for this group reflect missing lab measurements rather than intrinsic prognostic characteristics.

CRAB features (C: hypercalcemia; R: renal failure; A: anemia; or B: bone disease or bone pain) were assessed as markers of disease burden in MM. These features are widely used clinically to define MM disease severity and guide treatment initiation. The number of CRAB features present per patient was used as a proxy for disease burden and treatment trajectory. CRAB features were used as a complementary indicator of disease severity alongside ISS stage rather than as a standalone severity measure. Age (in years) was calculated relative to the index date from the patients’ date of birth. Patients with primary insurance healthcare coverage were identified as Medicare, Medicaid, Veterans Affairs, or private insurance types. Patient reported race was captured as White, Asian, Black or African American, Native Hawaiian or Pacific Islander, American Indian or Alaska Native, Multi Race, Other Race, or Unknown. Patients reported ethnicity was captured as Hispanic or Latino, Not Hispanic or Latino, or Unknown. To protect institutional confidentiality and patient privacy, individual UC location was identified as patients treated at UC-1, UC-2, or UC-3.

### 2.5. Statistical Analysis

Analyses were conducted between February and March 2025. Descriptive statistics were computed for each covariate and the overall cohort using ANOVA and chi-squared tests [31,32]. Associations with CAR-T receipt were modeled using a generalized linear model (GLM), adjusting for age, gender, ADI, primary insurance, UC location, number of CRAB features, ISS stage, race, and ethnicity. Odds ratios (ORs) with 95% confidence intervals (CIs) and *p*-values were reported to indicate the statistical significance of how likely a patient was to receive CAR-T therapy. Significance was defined as 95% CI did not span 1 and *p*-value < 0.05. Analyses were performed using R version 3.6.3. (R Project for Statistical Computing).

### 2.6. CAR-T Eligibility Identification Through Large Language Models

To identify CAR-T eligibility for patients from the UCSF deidentified clinical database, we applied similar inclusion and exclusion criteria described in the study population section. Additionally, patients without any associated clinical notes were excluded. Patients whose clinical notes had an index date on or before 1 January 2021, were excluded. We also excluded patients with clinical notes that were not within 365 days of the initiation date of their last cancer therapy or procedure. This resulted in a final cohort of 270 patients with 417 clinical notes (Supplement S2).

To identify CAR-T eligibility, we applied GPT-4 using a zero-shot learning approach with (temperature = 0). The model was prompted to analyze the “Assessment & Plan” section of the clinical notes written by physicians. The model was tasked with four objectives: (1) Was CAR-T discussed? [yes/no]. (2) Is the patient eligible for CAR-T? [yes/no/unclear]. (3) Provide the rationale for the eligibility determination. (4) Classify the eligibility rationale for CAR-T discussions into predefined groups: (“Ineligible due to disease criteria”, “Ineligible due to frailty or comorbidities”, “Ineligible due to social determinants of health”, “Ineligible due to not enough prior lines of therapy”, “Eligible now or potentially eligible in future”, “Not enough information to determine eligibility”, and “Other”). The model’s accuracy was tested and validated on a subsample of clinical notes by comparing its outputs against the ground truth to assess its performance in identifying CAR-T eligibility. GPT-4 model outputs were validated against clinician review and visualized. The identified CAR-T eligibility outputs were then visualized and presented in a tabular format.

## 3. Results

Our study included 12,360 patients (mean age 68.5 years, SD 12.8 years) with 320 receiving CAR-T (Table 1). A total of 51.6% of MM patients identified as Male, and 48.4% as Female. A total of 61.2% of patients identified as White, 7.8% Black or African American, and 14.9% as Hispanic or Latino. Patients treated at each UC location were primarily White, but UC-3 treated a higher proportion of Black and Unknown-race patients, while UC-1 had more Hispanic or Latino patients.

Primary insurance coverage was distributed as Medicaid (13.5%), Medicare (39.5%), Private (44.9%), and Veterans Affairs Insurance (2.1%). ISS stage distribution was ISS Stage I (65.3%), II (24.4%), III (2.8%), and None (7.5%). Patients treated at each UC location were primarily diagnosed with Stage I, but patients at UC-1 (21.4%) and UC-2 (39.2%) had a higher distribution of patients diagnosed with Stage II. Mean CRAB features per patient were 1.86 (SD 0.98), with anemia most common (Supplement S2).

Univariate analyses showed that race, ISS stage, socioeconomic status, and UC location were significant determinants of CAR-T receipt (Methods, *p* < 0.001, χ^2^ test) (Table 1). Regression adjusting for ISS stage, UC location, race, ethnicity, CRAB features, ADI, and primary insurance confirmed these associations (Table 2). Black or African American patients had lower odds of receiving CAR-T (OR = 0.33, 95% CI 0.17–0.62) compared to White patients in adjusted analysis, while Hispanic or Latino patients had slightly higher odds (OR = 1.26, 95% CI 0.87–1.81). These differences were consistent across treatment locations and disease severity categories. Higher CRAB feature count was associated with CAR-T receipt (OR = 1.43, 95% CI 1.27–1.62). Patients diagnosed with ISS Stage II (OR = 1.15, [95% CI, 0.89–1.48]) were more likely to receive a CAR-T treatment than patients with ISS Stage I MM (Figure 1).

**Figure 2:**
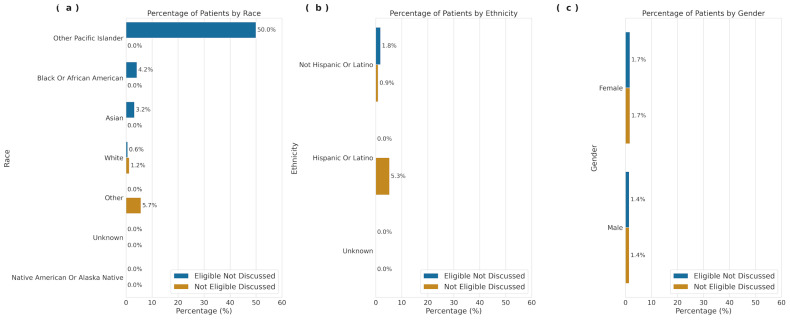
Distribution of UCSF multiple myeloma patients by (**a**) race, (**b**) ethnicity, and (**c**) gender, who were eligible for CAR-T therapy but did not have a discussion, and patients who had a discussion but were not eligible for CAR-T therapy.

UC location was a significant determinant of CAR-T treatment administration. Most CAR-T administration for MM patients was done at UC-2 (43.1%). Patients treated at UC-3 (OR = 0.42, [95% CI, 0.30–0.59]) were less likely to receive CAR-T therapy when compared to UC-1. We further investigated individual relationships between UC location, ISS stage, and race by performing an additional regression analysis to understand these differences and found significance for race at UC location (*p* < 0.001), ISS stage at UC location (*p* < 0.001), and number of CRAB features at UC location (*p* < 0.001). Interestingly, our results show that CAR-T treatment receipt is disparate among different population groups based on race, disease severity, and UC location (Figure 2). The distribution of patients within each UC location further explained why Black or African American patients and patients with ISS Stage I were less likely to receive CAR-T. (Supplement S2). The distribution showed that locations with fewer CAR-T treatments had a higher proportion of Black patients ([UC-1, n = 247], [UC-2, n = 291], [UC-3, n = 431]) and patients with ISS Stage I. This suggests that differences in CAR-T therapy utilization may be driven by patients’ access to locations more likely to offer therapy, rather than individual clinical decision-making.

**Figure 3:**
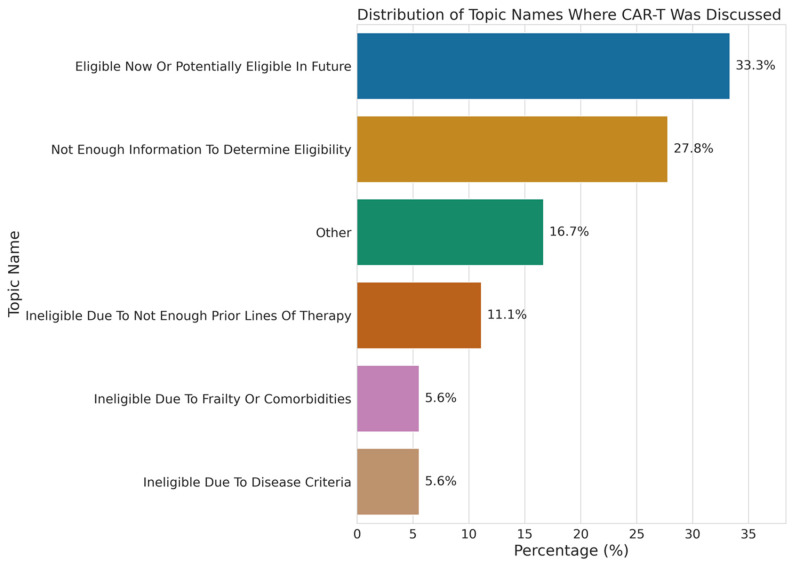
Distribution of topic modeling for UCSF multiple myeloma patients who had discussions about CAR-T therapy with their provider.

We deployed a large language model (GPT-4o) within a HIPAA-compliant environment to identify CAR-T eligibility for 270 UCSF patients whose clinical notes contained Assessment and Plan sections. We identified individuals who were considered eligible for CAR-T therapy based on eligibility guidelines but lacked documented discussions. Patients identified as Other Pacific Islander had the highest proportion of eligibility without discussion (50%), followed by Black or African American (4.2%), Asian (3.2%), and White (0.6%), highlighting potential differences in care where eligible patients are not being offered or engaged in discussions about CAR-T therapy (Figure 2). Among patients who had CAR-T discussions with their provider, 33.3% were classified as eligible now or potentially in the future, while 23.3% were ineligible (Figure 3).

## 4. Discussion

In this retrospective cohort study, we examined associations between disease characteristics, UC location, and demographics with receipt of CAR-T therapy among patients with MM within the UC Health system. We observed meaningful variation in CAR-T therapy receipt across UC treatment locations that offer CAR-T administration. Patients treated at UC-1 and UC-2 had higher rates of CAR-T therapy compared with those treated at UC-3. UC-1 and UC-2 function largely as specialty referral centers, whereas UC-3 provides a combination of primary and specialty care. These structural differences may contribute to variation in CAR-T utilization across sites. Differences in disease severity distributions across locations may also partially explain observed patterns.

Our model identified differences in CAR-T therapy receipt varied by patient age and race. Increasing age was associated with lower odds of receiving CAR-T therapy, consistent with current clinical practice, as older patients with relapsed or refractory MM may be preferentially treated with alternative therapies, such as bispecific antibodies, due to concerns regarding treatment-related toxicities including cytokine release syndrome (CRS), immune effector cell-associated neurotoxicity (ICANS), and delayed neurotoxicity such as Parkinsonism [33,34].

The observed association between patient-reported race and CAR-T therapy receipt warrants careful consideration. After adjustment for disease severity and treatment location, patients identifying as Black or African American remained less likely to receive CAR-T therapy than White patients. These findings are unlikely to reflect biological differences in treatment eligibility and instead may reflect structural factors influencing access to advanced therapies, including referral pathways, geographic proximity to CAR-T centers, caregiver requirements, and differences in care navigation. Prior studies in multiple myeloma have shown that when access to novel therapies is equitable, Black patients achieve similar or improved survival outcomes compared with White patients [3]. Our findings suggest that differences in CAR-T receipt may reflect inequitable access to innovative therapies rather than differences in clinical appropriateness.

Disease severity was also associated with CAR-T therapy receipt. Patients with a greater number of CRAB features, which more directly reflect disease burden and treatment trajectory, had higher odds of receiving CAR-T therapy. Although ISS stage was included as a baseline prognostic measure, relatively few patients were classified as ISS Stage III, and missing laboratory data limited interpretability for patients with unknown stage. Patients treated at UC-1 and UC-2 also had a higher proportion of early-stage diagnoses, which may contribute to higher CAR-T receipt at these sites.

Despite UC-3 treating a higher proportion of patients from minority populations and patients with lower neighborhood-level socioeconomic status, Area Deprivation Index (ADI) was not independently associated with CAR-T therapy receipt. This finding may reflect limitations of neighborhood-level measures in capturing individual-level socioeconomic barriers relevant to CAR-T eligibility and receipt [35,36]. The absence of statistical significance does not preclude clinical relevance, particularly for social factors such as caregiver availability, housing stability, or ability to travel, which are not fully captured by ADI. In contrast, patients identifying as Hispanic or Latino had higher odds of receiving CAR-T therapy compared with White patients, a finding that contrasts with prior reports of lower clinical trial participation in this population and warrants further investigation [10].

Using a large language model to analyze UCSF clinical notes, we identified patients who were considered eligible for CAR-T therapy but lacked a corresponding recorded discussion of treatment. This pattern was observed more frequently among patients identifying as Other Pacific Islander, Black or African American, and Asian. These findings suggest potential gaps in documentation, communication, or referral processes; however, they cannot distinguish between undocumented discussions, patient preferences, or other unmeasured barriers. Given the limited sample size and single-center note analysis, these results should be interpreted as exploratory. This approach provides partial insight into whether observed differences in CAR-T receipt reflect documented ineligibility versus absence of documented treatment discussions.

This study has several limitations. Changes in CAR-T indications and evolving clinical practice between 2021 and 2025 may introduce temporal bias, particularly when comparing utilization across centers. ISS staging was unavailable for a subset of patients due to missing beta-2 microglobulin measurements and limited cytogenetic data, which reduced statistical power and precluded use of the revised ISS. Although CRAB features were included as indicators of symptomatic disease burden, they reflect clinical manifestations of end-organ damage rather than comprehensive biological risk stratification and should therefore be interpreted as complementary markers rather than definitive measures of myeloma risk. Socioeconomic status was estimated using neighborhood-level ADI rather than individual-level measures, which may underestimate the contribution of social determinants to CAR-T receipt. In addition, analysis of CAR-T eligibility discussions was limited to patients with available UCSF clinical notes and could not fully capture undocumented clinical decision-making, referral pathways, or undocumented social factors. Finally, our ability to assess participation in clinical trials was limited, which may influence interpretation of CAR-T receipt patterns.

## 5. Conclusions

Receipt of CAR-T therapy among patients with MM within the UC Health system varied by treatment location, disease severity, and patient-reported race. These differences in CAR-T receipt persisted after adjustment for clinical and socioeconomic factors and occurred despite lower CAR-T receipt among populations that often experience worse survival outcomes. While race is an important indicator in this analysis, the observed patterns likely reflect broader structural and system-level factors related to care delivery, referral pathways, and trust in the healthcare system rather than biological differences. Because this study evaluates documented receipt rather than direct measures of access, further work incorporating individual-level social determinants of health, referral patterns, and clinical context will be critical for clarifying drivers of CAR-T utilization and promoting more equitable delivery of advanced therapies for patients with MM in different healthcare systems.

## Figures and Tables

**Table 1:** Baseline characteristics of multiple myeloma patients.

Did a Patient Receive CAR-T Therapy?			
Variable	Did Not Receive CAR-T Therapy	Received CAR-T Therapy	Total
	(N = 12,040)	(N = 320)	(N = 12,360)
Age			
Mean (SD)	68.7 (12.7)	63.1 (12.0)	68.5 (12.8)
Area Deprivation Index (ADI)			
Mean (SD)	3.88 (2.64)	4.28 (2.82)	3.89 (2.65)
Number of CRAB Features			
Mean (SD)	1.85 (0.976)	2.19 (0.904)	1.86 (0.975)
Gender			
Female	5852 (48.6%)	136 (42.5%)	5988 (48.4%)
Male	6188 (51.4%)	184 (57.5%)	6372 (51.6%)
Race			
American Indian or Alaska Native	51 (0.4%)	<10 (<10%)	53 (0.4%)
Asian	1062 (8.8%)	32 (10.0%)	1094 (8.9%)
Black or African American	959 (8.0%)	10 (3.1%)	969 (7.8%)
Native Hawaiian or Other Pacific Islander	46 (0.4%)	<10 (<10%)	47 (0.4%)
Other Race	1501 (12.5%)	66 (20.6%)	1567 (12.7%)
Unknown	1046 (8.7%)	14 (4.4%)	1060 (8.6%)
White	7375 (61.3%)	195 (60.9%)	7570 (61.2%)
Ethnicity			
Hispanic or Latino	1768 (14.7%)	78 (24.4%)	1846 (14.9%)
Not Hispanic or Latino	9305 (77.3%)	236 (73.8%)	9541 (77.2%)
Unknown	967 (8.0%)	<10 (<10%)	973 (7.9%)
ISS Stage			
I	7878 (65.4%)	199 (62.2%)	8077 (65.3%)
II	2903 (24.1%)	113 (35.3%)	3016 (24.4%)
III	336 (2.8%)	<10 (<10%)	343 (2.8%)
None	923 (7.7%)	<10 (<10%)	924 (7.5%)
UC Location			
UC-1	3442 (28.6%)	122 (38.1%)	3564 (28.8%)
UC-2	3419 (28.4%)	138 (43.1%)	3557 (28.8%)
UC-3	5179 (43.0%)	60 (18.8%)	5239 (42.4%)
Primary Insurance Coverage			
Medicaid	1611 (13.4%)	53 (16.6%)	1664 (13.5%)
Medicare	4775 (39.7%)	106 (33.1%)	4881 (39.5%)
Private	5394 (44.8%)	156 (48.8%)	5550 (44.9%)
VA Insurance	260 (2.2%)	<10 (<10%)	265 (2.1%)

Baseline characteristics of multiple myeloma patients with 1 or more MM therapies between January 2012 and May 2024.

**Table 2:** Results of logistic regression model.

Variable	Odds Ratio (95%CI)	*p*-Value
Area Deprivation Index (ADI)	1.02 (0.97–1.07)	0.395
Age	0.97 (0.96–0.98)	<0.001
UC Location		
UC-2	1.42 (1.09–1.87)	0.011
UC-3	0.42 (0.30–0.59)	<0.001
International Staging System (ISS)		
II	1.15 (0.89–1.48)	0.271
III	0.69 (0.32–1.50)	0.348
None	0.07 (0.01–0.48)	0.007
Number of CRAB Features	1.43 (1.27–1.62)	<0.001
Race		
American Indian or Alaska Native	1.04 (0.24–4.43)	0.959
Asian	0.99 (0.67–1.46)	0.959
Black or African American	0.33 (0.17–0.62)	<0.001
Native Hawaiian or Other Pacific Islander	0.77 (0.1–5.72)	0.802
Other	1.07 (0.73–1.56)	0.736
Unknown	1.26 (0.67–2.37)	0.47
Ethnicity		
Hispanic or Latino	1.26 (0.87–1.81)	0.216
Unknown	0.36 (0.15–0.88)	0.026
Primary Insurance Coverage		
Medicaid	0.71 (0.50–1.01)	0.056
Medicare	0.96 (0.73–1.25)	0.752
Veteran Affairs Insurance	0.61 (0.25–1.53)	0.292

Odds ratio table of patients receiving CAR-T therapies across patient characteristics. An OR = 1 indicates no difference between receiving or not receiving CAR-T therapies, OR > 1 indicates increased likelihood of receiving a CAR-T therapy, and OR < 1 indicates decreased likelihood of receiving CAR-T as a therapy.

## Data Availability

The data supporting this study are restricted due to the sensitivity of deidentified patient data. Access is not available upon request.

## Supplementary Materials

The following supporting information can be downloaded at: https://www.mdpi.com/article/10.3390/cancers18040669/s1, Supplement S1: Section S1. Diagnosis codes for multiple myeloma patients; Section S2. RxNorm codes for multiple myeloma patients; Section S3. Procedural codes for multiple myeloma patients. Supplement S2: Section S1. UCHDW cohort selection; Section S2. Distribution of multiple myeloma patients based on baseline demographics; Section S3. UCSF cohort selection; Section S4. UCSF deidentified clinical notes prompt and identification.
