# Supplementary material for: Investigating CAR-T Treatment Access for Multiple Myeloma Patients Using Real-World Evidence
Source: Cancers (Basel). 2026 Feb 18;18(4):669. doi: 10.3390/cancers18040669 (PMC12938920; doi:10.3390/cancers18040669)
Supplement: Supplementary file 1 [file cancers-18-00669-s001.zip › MM Supplement_S1_final.pdf]

Supplement S1 Online Content

Jaysón Davidson et al. Investigating CAR-T Treatment Access for Multiple Myeloma Patients Using Real-World Evidence

Methods.
Section S1. Diagnosis Codes for Multiple Myeloma Patients
Section S2. RxNorm Codes for Multiple Myeloma Patients
Section S3. Procedural Codes for Multiple Myeloma Patients

Section S1. Diagnosis Codes for Multiple Myeloma Patients

Table describing diagnosis codes for Multiple Myeloma patients who received cancer therapies or procedures at a given UC health site based on the inclusion criteria.

S1.1 Table S1: Diagnosis Codes for Multiple Myeloma Patients

**Diagnosis Codes**	**Code Type**	**Diagnosis**
**109983007**	SNOMED	Monoclonal gammopathy (clinical)
**109989006**	SNOMED	Multiple myeloma
**95209008**	SNOMED	Plasma cell leukemia in remission
**415111003**	SNOMED	Plasma cell neoplasm
**122981000119101**	SNOMED	Plasma cell leukemia in relapse
**C90.00**	ICD-10	Multiple myeloma not having achieved remission
**C90.01**	ICD-10	Multiple myeloma in remission
**C90.02**	ICD-10	Multiple myeloma in relapse

Section S2. RxNorm Codes for Multiple Myeloma Patients

Table describing RxNorm codes for Multiple Myeloma patients who received cancer therapies.

S2.1 Table S2: RxNorm Codes for Multiple Myeloma Patients

**Drug Codes**	**Code Type**	**MM Drugs**
**212994**	RxNorm	carmustine 7.7 MG Drug Implant [Gliadel]
**1369734**	RxNorm	pomalidomide 4 MG Oral Capsule
**1303132**	RxNorm	prednisone 2 MG Delayed Release Oral Tablet
**205301**	RxNorm	prednisone 5 MG/ML Oral Solution
**197580**	RxNorm	dexamethasone 1.5 MG Oral Tablet
**1790127**	RxNorm	25 ML doxorubicin hydrochloride liposome 2 MG/ML Injection
**2548733**	RxNorm	{4 (selinexor 60 MG Oral Tablet) } Pack
**373585**	RxNorm	prednisone Oral Tablet
**2536443**	RxNorm	70 ML idecabtagene vicleucel 6571428 CELLS/ML Injection
**309679**	RxNorm	dexamethasone 0.001 MG/MG / neomycin 0.0035 MG/MG / polymyxin B 10 UNT/MG Ophthalmic Ointment
**2536451**	RxNorm	100 ML idecabtagene vicleucel 4600000 CELLS/ML Injection [Abecma]
**2594780**	RxNorm	70 ML ciltacabtagene autoleucel 1430000 CELLS/ML Injection
**763179**	RxNorm	{48 (prednisone 5 MG Oral Tablet) } Pack
**763183**	RxNorm	{48 (prednisone 10 MG Oral Tablet) } Pack
**2644888**	RxNorm	elranatamab-bcmm 40 MG/ML [Elrexfio]
**1303135**	RxNorm	prednisone 5 MG Delayed Release Oral Tablet
**759697**	RxNorm	{35 (dexamethasone 1.5 MG Oral Tablet) } Pack
**205669**	RxNorm	dexamethasone 1 MG/ML Ophthalmic Suspension [Maxidex]
**1726113**	RxNorm	elotuzumab Injection [Empliciti]
**312615**	RxNorm	prednisone 20 MG Oral Tablet
**1790115**	RxNorm	10 ML doxorubicin hydrochloride liposome 2 MG/ML Injection
**1242231**	RxNorm	lenalidomide 2.5 MG Oral Capsule
**200390**	RxNorm	thalidomide 50 MG Oral Capsule
**1790095**	RxNorm	doxorubicin hydrochloride 50 MG Injection

**1809548**	RxNorm	hyaluronidase Injection
**1723770**	RxNorm	ixazomib 4 MG Oral Capsule
**1812073**	RxNorm	dexamethasone Injection
**1721957**	RxNorm	daratumumab 20 MG/ML Injection
**1805015**	RxNorm	0.5 ML bendamustine hydrochloride 90 MG/ML Injection
**1812095**	RxNorm	1 ML dexamethasone phosphate 4 MG/ML Prefilled Syringe
**2282030**	RxNorm	isatuximab-irfc 20 MG/ML Injection [Sarclisa]
**763181**	RxNorm	{21 (prednisone 5 MG Oral Tablet) } Pack
**1805001**	RxNorm	bendamustine hydrochloride 100 MG Injection
**1191138**	RxNorm	doxorubicin hydrochloride 2 MG/ML Injectable Solution
**309690**	RxNorm	dexamethasone phosphate 0.0005 MG/MG Ophthalmic Ointment
**2375143**	RxNorm	daratumumab-fihj 120 MG/ML / hyaluronidase-fihj 2000 UNT/ML Injection [Darzalex Faspro]
**1986445**	RxNorm	tisagenlecleucel Injection [Kymriah]
**1987407**	RxNorm	68 ML axicabtagene ciloleucel 2940000 CELLS/ML Injection [Yescarta]
**198146**	RxNorm	prednisone 2.5 MG Oral Tablet
**2536453**	RxNorm	idecabtagene vicleucel 4600000 CELLS/ML Injection [Abecma]
**2178396**	RxNorm	{12 (selinexor 20 MG Oral Tablet) } Pack
**1369718**	RxNorm	pomalidomide 1 MG Oral Capsule
**2282031**	RxNorm	25 ML isatuximab-irfc 20 MG/ML Injection
**3002**	RxNorm	cyclophosphamide
**1806942**	RxNorm	carfilzomib 60 MG Injection [Kyprolis]
**2548762**	RxNorm	selinexor 40 MG Oral Tablet
**2375136**	RxNorm	15 ML daratumumab-fihj 120 MG/ML / hyaluronidase-fihj 2000 UNT/ML Injection
**1303125**	RxNorm	prednisone 1 MG Delayed Release Oral Tablet
**1927892**	RxNorm	hyaluronidase, human recombinant 2000 UNT/ML / rituximab 120 MG/ML Injection [Rituxan Hycela]
**1437968**	RxNorm	cyclophosphamide 25 MG Oral Capsule
**2382607**	RxNorm	hyaluronidase / pertuzumab / trastuzumab Injection [Phesgo]
**1745090**	RxNorm	melphalan 50 MG Injection [Evomela]
**368873**	RxNorm	melphalan Oral Tablet [Alkeran]
**402244**	RxNorm	bortezomib 3.5 MG Injection [Velcade]
**2644890**	RxNorm	elranatamab Injection [Elrexfio]
**2548738**	RxNorm	selinexor 50 MG Oral Tablet
**403908**	RxNorm	ciprofloxacin 3 MG/ML / dexamethasone 1 MG/ML Otic Suspension
**2387350**	RxNorm	brexucabtagene autoleucel Injection [Tecartus]
**2594785**	RxNorm	ciltacabtagene autoleucel Injection [Carvykti]
**2479143**	RxNorm	lisocabtagene maraleucel Injection [Breyanzi]
**2479147**	RxNorm	lisocabtagene maraleucel 70000000 CELLS/ML Injection [Breyanzi]
**2594779**	RxNorm	ciltacabtagene autoleucel Injection
**2479140**	RxNorm	4.6 ML lisocabtagene maraleucel 70000000 CELLS/ML Injection
**2594789**	RxNorm	30 ML ciltacabtagene autoleucel 3330000 CELLS/ML Injection
**2536449**	RxNorm	100 ML idecabtagene vicleucel 4600000 CELLS/ML Injection
**1987402**	RxNorm	68 ML axicabtagene ciloleucel 2940000 CELLS/ML Injection
**1986442**	RxNorm	tisagenlecleucel 250000000 CELLS Injection
**2044973**	RxNorm	tisagenlecleucel 600000000 CELLS Injection
**2572002**	RxNorm	68 ML brexucabtagene autoleucel 1471000 CELLS/ML Injection
**2044974**	RxNorm	tisagenlecleucel 600000000 CELLS [Kymriah]
**2387347**	RxNorm	68 ML brexucabtagene autoleucel 2940000 CELLS/ML Injection
**2536434**	RxNorm	30 ML idecabtagene vicleucel 15333333 CELLS/ML Injection
**643712**	RxNorm	lenalidomide 15 MG Oral Capsule
**1812194**	RxNorm	1 ML dexamethasone phosphate 4 MG/ML Injection
**208813**	RxNorm	dexamethasone 1 MG/ML / tobramycin 3 MG/ML Ophthalmic Suspension [Tobradex]
**2282023**	RxNorm	5 ML isatuximab-irfc 20 MG/ML Injection
**2601544**	RxNorm	bortezomib 2.5 MG Injection
**1734917**	RxNorm	cyclophosphamide 500 MG Injection
**197549**	RxNorm	cyclophosphamide 25 MG Oral Tablet
**1805013**	RxNorm	bendamustine hydrochloride 90 MG/ML Injection

**8640**	RxNorm	prednisone
**197919**	RxNorm	melphalan 2 MG Oral Tablet
**828703**	RxNorm	1.2 ML plerixafor 20 MG/ML Injection [Mozobil]
**198148**	RxNorm	prednisone 50 MG Oral Tablet
**2619437**	RxNorm	teclistamab Injection [Tecvayli]
**309696**	RxNorm	dexamethasone phosphate 10 MG/ML Injectable Solution
**1369730**	RxNorm	pomalidomide 3 MG Oral Capsule
**312617**	RxNorm	prednisone 5 MG Oral Tablet
**1723757**	RxNorm	ixazomib 2.3 MG Oral Capsule
**700416**	RxNorm	thalidomide 150 MG Oral Capsule
**596881**	RxNorm	carmustine 3.3 MG/ML Injectable Solution [BiCNU]
**309680**	RxNorm	dexamethasone 1 MG/ML / neomycin 3.5 MG/ML / polymyxin B 10000 UNT/ML Ophthalmic Suspension
**1806940**	RxNorm	carfilzomib 60 MG Injection
**2548732**	RxNorm	selinexor 60 MG Oral Tablet
**1869595**	RxNorm	{27 (dexamethasone 1.5 MG Oral Tablet) } Pack
**309686**	RxNorm	dexamethasone 0.1 MG/ML Oral Solution
**1799310**	RxNorm	doxorubicin hydrochloride 2 MG/ML Injectable Solution [Adriamycin]
**1428947**	RxNorm	lenalidomide 20 MG Oral Capsule
**1726096**	RxNorm	bendamustine hydrochloride 25 MG/ML
**2548763**	RxNorm	{8 (selinexor 40 MG Oral Tablet) } Pack
**1114695**	RxNorm	bendamustine hydrochloride 5 MG/ML Injectable Solution
**846192**	RxNorm	{21 (dexamethasone 1.5 MG Oral Tablet) } Pack
**854177**	RxNorm	dexamethasone 0.7 MG Drug Implant
**2568661**	RxNorm	5 ML cyclophosphamide 200 MG/ML Injection
**198145**	RxNorm	prednisone 10 MG Oral Tablet
**309683**	RxNorm	dexamethasone 1 MG/ML / tobramycin 3 MG/ML Ophthalmic Suspension
**2099700**	RxNorm	1 ML dexamethasone phosphate 10 MG/ML Prefilled Syringe
**197579**	RxNorm	dexamethasone 1 MG Oral Tablet
**1805011**	RxNorm	2 ML bendamustine hydrochloride 90 MG/ML Injection
**1734340**	RxNorm	etoposide 100 MG Injection
**1806936**	RxNorm	carfilzomib Injection [Kyprolis]
**1734921**	RxNorm	cyclophosphamide 2000 MG Injection
**2644447**	RxNorm	talquetamab
**1740949**	RxNorm	melphalan Injection
**1806934**	RxNorm	carfilzomib 30 MG Injection
**1790094**	RxNorm	doxorubicin Injection
**2531374**	RxNorm	melphalan flufenamide 20 MG Injection
**2386859**	RxNorm	cyclophosphamide 200 MG/ML Injectable Solution
**2387843**	RxNorm	belantamab mafodotin-blmf 100 MG Injection [Blenrep]
**2178395**	RxNorm	selinexor 20 MG Oral Tablet
**1804994**	RxNorm	bortezomib Injection
**828700**	RxNorm	1.2 ML plerixafor 20 MG/ML Injection
**2387838**	RxNorm	belantamab mafodotin-blmf 100 MG Injection
**197550**	RxNorm	cyclophosphamide 50 MG Oral Tablet
**2644457**	RxNorm	talquetamab Injection [Talvey]
**1721958**	RxNorm	daratumumab 20 MG/ML Injection [Darzalex]
**2108015**	RxNorm	dexamethasone 0.4 MG Drug Implant
**309684**	RxNorm	dexamethasone 1 MG/ML Oral Solution
**360176**	RxNorm	thalidomide 100 MG Oral Capsule
**2178406**	RxNorm	{32 (selinexor 20 MG Oral Tablet) } Pack
**1734916**	RxNorm	cyclophosphamide Injection
**1805004**	RxNorm	bendamustine hydrochloride 100 MG Injection [Treanda]
**309692**	RxNorm	dexamethasone 1 MG/ML Ophthalmic Suspension
**310248**	RxNorm	etoposide 20 MG/ML Injectable Solution
**2108020**	RxNorm	dexamethasone 0.4 MG Drug Implant [Dextenza]
**198144**	RxNorm	prednisone 1 MG Oral Tablet

**208821**	RxNorm	dexamethasone 0.001 MG/MG / tobramycin 0.003 MG/MG Ophthalmic Ointment [Tobradex]
**402243**	RxNorm	bortezomib 3.5 MG Injection
**1721951**	RxNorm	5 ML daratumumab 20 MG/ML Injection
**1812079**	RxNorm	1 ML dexamethasone phosphate 10 MG/ML Injection
**1437969**	RxNorm	cyclophosphamide 50 MG Oral Capsule
**849115**	RxNorm	dexamethasone 0.5 MG/ML / tobramycin 3 MG/ML Ophthalmic Suspension
**226343**	RxNorm	dexamethasone phosphate 1 MG/ML Ophthalmic Solution
**2375141**	RxNorm	15 ML daratumumab-fihj 120 MG/ML / hyaluronidase-fihj 2000 UNT/ML Injection [Darzalex Faspro]
**208591**	RxNorm	dexamethasone 1 MG/ML / neomycin 3.5 MG/ML / polymyxin B 10000 UNT/ML Ophthalmic Suspension [Maxitrol]
**1726102**	RxNorm	bendamustine hydrochloride 25 MG/ML Injectable Solution [Bendeka]
**2121587**	RxNorm	{39 (dexamethasone 1.5 MG Oral Tablet) } Pack
**1943550**	RxNorm	{49 (dexamethasone 1.5 MG Oral Tablet) } Pack
**197576**	RxNorm	dexamethasone 0.25 MG Oral Tablet
**2619431**	RxNorm	teclistamab Injection
**2178408**	RxNorm	{20 (selinexor 20 MG Oral Tablet) } Pack
**2548739**	RxNorm	{8 (selinexor 50 MG Oral Tablet) } Pack
**2382989**	RxNorm	{24 (selinexor 20 MG Oral Tablet) } Pack
**370630**	RxNorm	hyaluronidase Injectable Solution
**208601**	RxNorm	dexamethasone 0.001 MG/MG / neomycin 0.0035 MG/MG / polymyxin B 10 UNT/MG Ophthalmic Ointment [Maxitrol]
**309682**	RxNorm	dexamethasone 0.001 MG/MG / tobramycin 0.003 MG/MG Ophthalmic Ointment
**1790116**	RxNorm	doxorubicin hydrochloride liposome 2 MG/ML Injection
**2548768**	RxNorm	{4 (selinexor 40 MG Oral Tablet) } Pack
**2178266**	RxNorm	bendamustine hydrochloride 25 MG/ML Injectable Solution [Belrapzo]
**1790099**	RxNorm	10 ML doxorubicin hydrochloride 2 MG/ML Injection
**197577**	RxNorm	dexamethasone 0.5 MG Oral Tablet
**643720**	RxNorm	lenalidomide 25 MG Oral Capsule
**854181**	RxNorm	dexamethasone 0.7 MG Drug Implant [Ozurdex]
**1116927**	RxNorm	dexamethasone phosphate 4 MG/ML Injectable Solution
**1805007**	RxNorm	bendamustine hydrochloride 25 MG Injection
**360177**	RxNorm	thalidomide 200 MG Oral Capsule
**1790098**	RxNorm	doxorubicin hydrochloride 2 MG/ML Injection
**309012**	RxNorm	carmustine 100 MG Injection
**1369726**	RxNorm	pomalidomide 2 MG Oral Capsule
**2046642**	RxNorm	carfilzomib 10 MG Injection
**1790129**	RxNorm	doxorubicin hydrochloride liposome 2 MG/ML Injection [Doxil]
**1726439**	RxNorm	20 ML daratumumab 20 MG/ML Injection
**197583**	RxNorm	dexamethasone 6 MG Oral Tablet
**315187**	RxNorm	prednisone 1 MG/ML Oral Solution
**1374372**	RxNorm	dexamethasone acetate 8 MG/ML Injectable Suspension
**2108345**	RxNorm	dexamethasone Metered Dose Inhaler
**2382992**	RxNorm	{8 (selinexor 20 MG Oral Tablet) } Pack
**1790103**	RxNorm	doxorubicin hydrochloride 10 MG Injection
**602910**	RxNorm	lenalidomide 10 MG Oral Capsule
**1723765**	RxNorm	ixazomib 3 MG Oral Capsule
**763185**	RxNorm	{21 (prednisone 10 MG Oral Tablet) } Pack
**1790100**	RxNorm	25 ML doxorubicin hydrochloride 2 MG/ML Injection
**790699**	RxNorm	bendamustine Injectable Solution [Treanda]
**2619441**	RxNorm	1.7 ML teclistamab-cqyv 90 MG/ML Injection
**2619432**	RxNorm	3 ML teclistamab-cqyv 10 MG/ML Injection
**2178404**	RxNorm	{16 (selinexor 20 MG Oral Tablet) } Pack
**309695**	RxNorm	dexamethasone 1 MG/ML / neomycin 3.5 MG/ML Ophthalmic Solution
**2531379**	RxNorm	melphalan flufenamide 20 MG Injection [Pepaxto]
**3264**	RxNorm	dexamethasone
**343033**	RxNorm	dexamethasone 0.75 MG Oral Tablet

**197581**	RxNorm	dexamethasone 2 MG Oral Tablet
**1790097**	RxNorm	5 ML doxorubicin hydrochloride 2 MG/ML Injection
**309013**	RxNorm	carmustine 7.7 MG Drug Implant
**759481**	RxNorm	{51 (dexamethasone 1.5 MG Oral Tablet) } Pack
**1249710**	RxNorm	dexamethasone sodium phosphate 0.2 MG/ML Injectable Solution
**197582**	RxNorm	dexamethasone 4 MG Oral Tablet
**311487**	RxNorm	melphalan 50 MG Injection
**1734919**	RxNorm	cyclophosphamide 1000 MG Injection
**206831**	RxNorm	etoposide 20 MG/ML Injectable Solution [Toposar]
**1249712**	RxNorm	dexamethasone sodium phosphate 0.4 MG/ML Injectable Solution
**2261802**	RxNorm	dexamethasone 20 MG Oral Tablet
**1790178**	RxNorm	doxorubicin hydrochloride liposome 2 MG/ML Injection [LipoDox]
**1726097**	RxNorm	bendamustine hydrochloride 25 MG/ML Injectable Solution
**197687**	RxNorm	etoposide 50 MG Oral Capsule
**602912**	RxNorm	lenalidomide 5 MG Oral Capsule

Section S3. Procedural Codes for Multiple Myeloma Patients

Table describing procedural codes for Multiple Myeloma patients who undergone a cancer procedure.

S3.1 Table S3: Procedural Codes for Multiple Myeloma Patients

**Procedure Codes**	**Code Type**	**MM Procedures**
**77761**	CPT4	Intracavitary radiation source application; simple
**77338**	CPT4	Multi-leaf collimator (MLC) device(s) for intensity modulated radiation therapy (IMRT), design and construction per IMRT plan
**77423**	CPT4	High energy neutron radiation treatment delivery, 1 or more isocenter(s) with coplanar or non-coplanar geometry with blocking and/or wedge, and/or compensator(s)
**77777**	CPT4	Interstitial radiation source application; intermediate
**49412**	CPT4	Placement of interstitial device(s) for radiation therapy guidance (eg, fiducial markers, dosimeter), open, intra-abdominal, intrapelvic, and/or retroperitoneum, including image guidance, if performed, single or multiple (List separately in addition to co
**61770**	CPT4	Stereotactic localization, including burr hole(s), with insertion of catheter(s) or probe(s) for placement of radiation source
**76145**	CPT4	Medical physics dose evaluation for radiation exposure that exceeds institutional review threshold, including report
**55876**	CPT4	Placement of interstitial device(s) for radiation therapy guidance (eg, fiducial markers, dosimeter), prostate (via needle, any approach), single or multiple
**49327**	CPT4	Laparoscopy, surgical; with placement of interstitial device(s) for radiation therapy guidance (eg, fiducial markers, dosimeter), intra-abdominal, intrapelvic, and/or retroperitoneum, including imaging guidance, if performed, single or multiple (List sepa
**77763**	CPT4	Intracavitary radiation source application; complex
**77373**	CPT4	Stereotactic body radiation therapy, treatment delivery, per fraction to 1 or more lesions, including image guidance, entire course not to exceed 5 fractions
**77014**	CPT4	Computed tomography guidance for placement of radiation therapy fields
**1922**	CPT4	Anesthesia for non-invasive imaging or radiation therapy
**77470**	CPT4	Special treatment procedure (eg, total body irradiation, hemibody radiation, per oral or endocavitary irradiation)
**67218**	CPT4	Destruction of localized lesion of retina (eg, macular edema, tumors), 1 or more sessions; radiation by implantation of source (includes removal of source)
**0190T**	CPT4	Placement of intraocular radiation source applicator (List separately in addition to primary procedure)
**57156**	CPT4	Insertion of a vaginal radiation afterloading apparatus for clinical brachytherapy
**77300**	CPT4	Basic radiation dosimetry calculation, central axis depth dose calculation, TDF, NSD, gap calculation, off axis factor, tissue inhomogeneity factors, calculation of non-ionizing radiation surface and depth dose, as required during course of treatment, onl
**77421**	CPT4	Stereoscopic X-ray guidance for localization of target volume for the delivery of radiation therapy

**77778**	CPT4	Interstitial radiation source application, complex, includes supervision, handling, loading of radiation source, when performed
**77776**	CPT4	Interstitial radiation source application; simple
**92974**	CPT4	Transcatheter placement of radiation delivery device for subsequent coronary intravascular brachytherapy (List separately in addition to code for primary procedure)
**0197T**	CPT4	Intra-fraction localization and tracking of target or patient motion during delivery of radiation therapy (eg, 3D positional tracking, gating, 3D surface tracking), each fraction of treatment
**32553**	CPT4	Placement of interstitial device(s) for radiation therapy guidance (eg, fiducial markers, dosimeter), percutaneous, intra-thoracic, single or multiple
**32701**	CPT4	Thoracic target(s) delineation for stereotactic body radiation therapy (SRS/SBRT), (photon or particle beam), entire course of treatment
**77432**	CPT4	Stereotactic radiation treatment management of cranial lesion(s) (complete course of treatment consisting of 1 session)
**77399**	CPT4	Unlisted procedure, medical radiation physics, dosimetry and treatment devices, and special services
**77469**	CPT4	Intraoperative radiation treatment management
**77762**	CPT4	Intracavitary radiation source application; intermediate
**19294**	CPT4	Preparation of tumor cavity, with placement of a radiation therapy applicator for intraoperative radiation therapy (IORT) concurrent with partial mastectomy (List separately in addition to code for primary procedure)
**77435**	CPT4	Stereotactic body radiation therapy, treatment management, per treatment course, to 1 or more lesions, including image guidance, entire course not to exceed 5 fractions
**77425**	CPT4	Intraoperative radiation treatment delivery, electrons, single treatment session
**77424**	CPT4	Intraoperative radiation treatment delivery, x-ray, single treatment session
**77385**	CPT4	Intensity modulated radiation treatment delivery (IMRT), includes guidance and tracking, when performed; simple
**77386**	CPT4	Intensity modulated radiation treatment delivery (IMRT), includes guidance and tracking, when performed; complex
**77387**	CPT4	Guidance for localization of target volume for delivery of radiation treatment, includes intrafraction tracking, when performed
**76950**	CPT4	Ultrasonic guidance for placement of radiation therapy fields
**49411**	CPT4	Placement of interstitial device(s) for radiation therapy guidance (eg, fiducial markers, dosimeter), percutaneous, intra-abdominal, intra-pelvic (except prostate), and/or retroperitoneum, single or multiple
**G6017**	HCPCS	Intra-fraction localization and tracking of target or patient motion during delivery of radiation therapy (eg,3d positional tracking, gating, 3d surface tracking), each fraction of treatment
**C9726**	HCPCS	Placement and removal (if performed) of applicator into breast for intraoperative radiation therapy, add-on to primary breast procedure
**C9728**	HCPCS	Placement of interstitial device(s) for radiation therapy/surgery guidance (e.g., fiducial markers, dosimeter), for other than the following sites (any approach): abdomen, pelvis, prostate, retroperitoneum, thorax, single or multiple
**S2270**	HCPCS	Insertion of vaginal cylinder for application of radiation source or clinical brachytherapy (report separately in addition to radiation source delivery) (Deprecated)
**G6001**	HCPCS	Ultrasonic guidance for placement of radiation therapy fields
**41.04**	ICD9Proc	Autologous hematopoietic stem cell transplant without purging
**92.26**	ICD9Proc	Teleradiotherapy of other particulate radiation
**41.07**	ICD9Proc	Autologous hematopoietic stem cell transplant with purging
**41.08**	ICD9Proc	Allogeneic hematopoietic stem cell transplant with purging
**92.41**	ICD9Proc	Intra-operative electron radiation therapy
**5304008**	SNOMED	Deep radiation therapy, 200-300 KVP
**10611004**	SNOMED	External beam radiation therapy protons
**441799006**	SNOMED	Intensity modulated radiation therapy
**446253009**	SNOMED	Allogeneic bone marrow transplantation with purging
**448461009**	SNOMED	Stereotactic radiation therapy using megavoltage radiation
**372049008**	SNOMED	Assessment of history of previous radiation exposure
**45643008**	SNOMED	External beam radiation therapy using electrons
**19944001**	SNOMED	Autologous bone marrow transplant without purging
**64522003**	SNOMED	Destruction of chorioretinal lesion by implantation of radiation source
**445757003**	SNOMED	Allogeneic bone marrow transplantation without purging
**85257004**	SNOMED	Destruction of chorioretinal lesion by radiation therapy
