# Supplementary material for: Investigating CAR-T Treatment Access for Multiple Myeloma Patients Using Real-World Evidence
Source: Cancers (Basel). 2026 Feb 18;18(4):669. doi: 10.3390/cancers18040669 (PMC12938920; doi:10.3390/cancers18040669)
Supplement: Supplementary file 1 [file cancers-18-00669-s001.zip › MM Supplement_S2_final.pdf]

Supplement S2 Online Content

Jaysón Davidson et al. Investigating CAR-T Treatment Access for Multiple Myeloma Patients Using Real-World Evidence

Methods.
Section S1. Cohort Selection
Section S2. Distribution of Multiple Myeloma Patients based on Baseline Demographics
Section S3. UCSF Cohort Selection
Section S4. UCSF Deidentified Clinical Notes Prompt and Identification

Section S1. Cohort Selection

Flow diagram represents a STROBE chart indicating number of Multiple Myeloma patients who received cancer therapies or procedures at a given UC health site based on the inclusion criteria.

S1.1 Figure S1: Cohort selection for Multiple Myeloma Patients

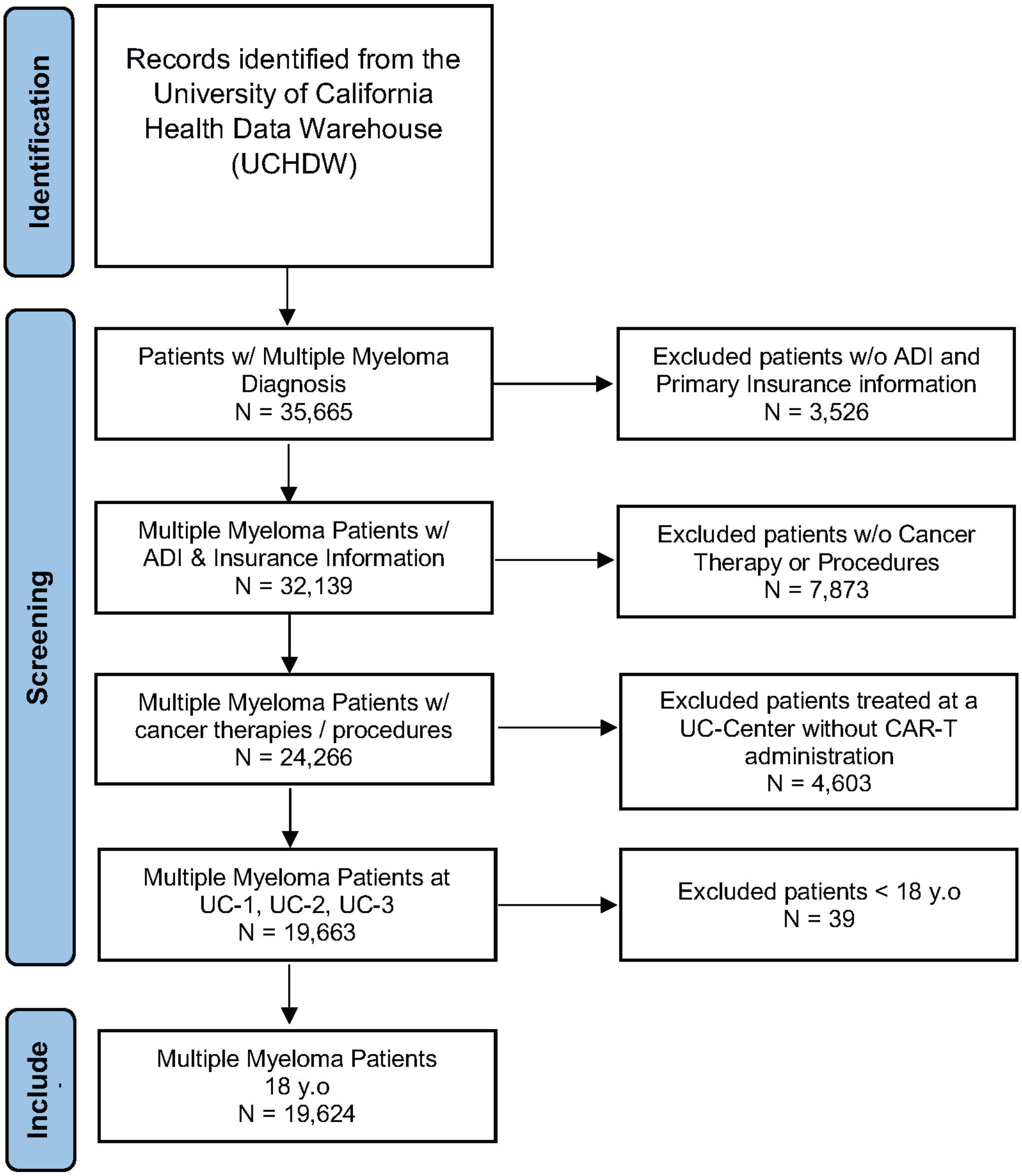

Section S2. Distribution of Multiple Myeloma Patients based on Baseline Demographics

Each table represents the distribution of multiple myeloma patients based on their disease severity, race, and UC-location.

Table S1: Distribution of Race by UC-Location

Race	UC-1	UC-2	UC-3
**American Indian or Alaska Native**	15	18	20
**Asian**	291	398	405
**Black or African American**	247	291	431
**Native Hawaiian or Other Pacific Islander**	13	19	15
**Other Race**	859	387	321
**Unknown**	67	110	883
**White**	2072	2334	3164

Table S2: Distribution of Ethnicity by UC-Location

Ethnicity	UC-1	UC-2	UC-3
**Hispanic or Latino**	788	392	666
**Not Hispanic or Latino**	2690	3017	3837
**Unknown**	86	151	737

Table S3: Distribution of International Staging System (ISS) Disease Stage by UC-Location

ISS Disease Stage	UC-1	UC-2	UC-3
**I**	2453	1778	3846
**II**	762	1394	860
**III**	73	77	193
**None**	276	308	340

Table S4: Distribution of Number of CRAB Features by UC-Location

Number of CRAB Features	UC-1	UC-2	UC-3
**0**	73	354	230
**1**	1142	1364	1786
**2**	1314	1083	1801
**3**	873	582	1154
**4**	160	169	262
**5**	<10	<10	<10

Table S5: Distribution of CRAB Features

CRAB Feature	Counts
**Anemia**	5779
**Renal Insufficiency**	3923
**HyperCalcemia**	1286
**Bone Lesion**	0

Section S3. UCSF Cohort Selection

Flow diagram represents a STROBE chart indicating number of Multiple Myeloma patients with clinical notes who received cancer therapies or procedures at the UCSF health site based on the inclusion criteria.

S3.1 Figure S2: Cohort selection for Multiple Myeloma Patients

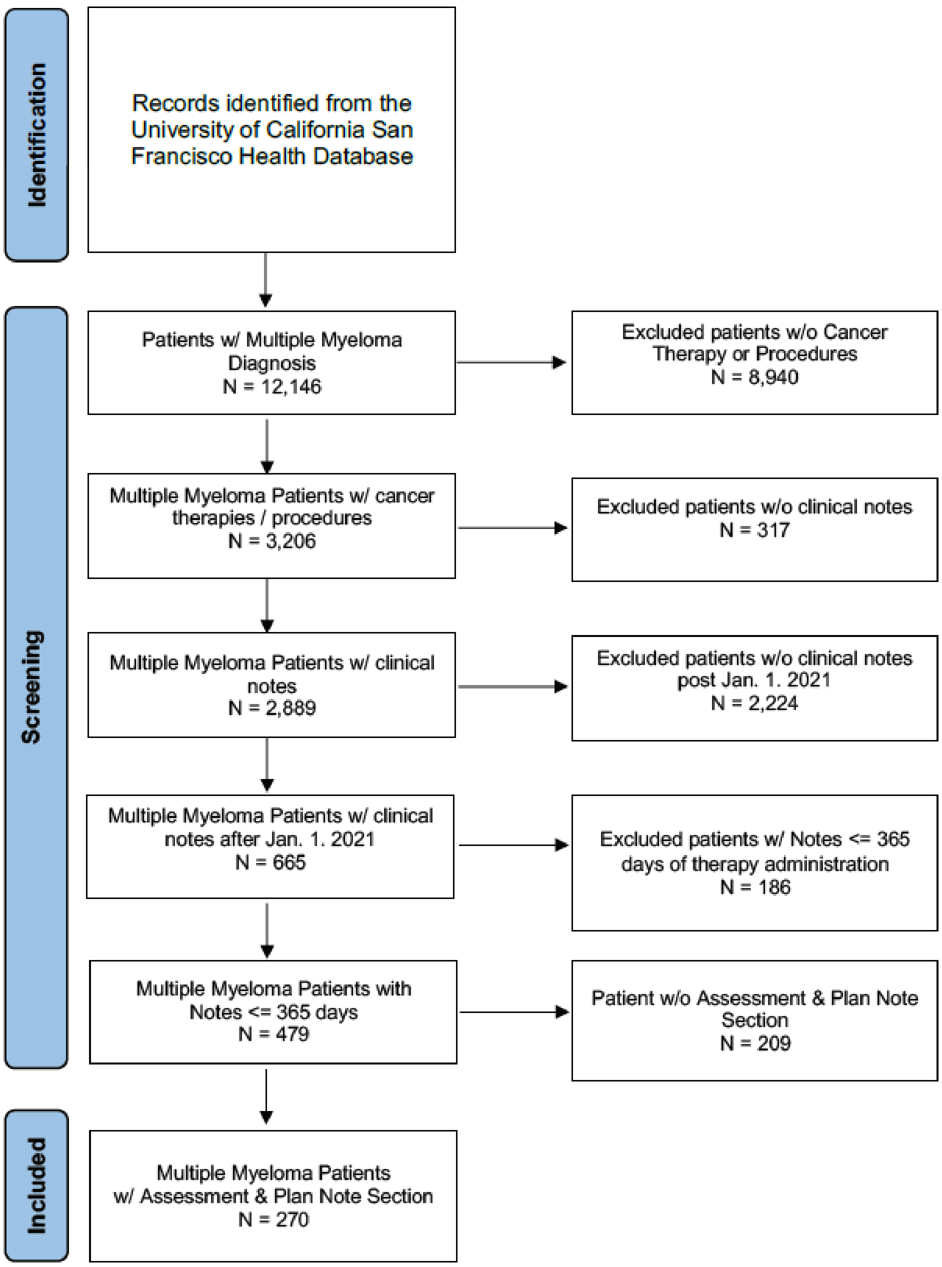

Section S4. UC-2 Deidentified Clinical Notes Prompt and Identification

UC-2 Deidentified Clinical Note Dataset utilized for CAR-T Eligibility Identification with GPT-4 Prompt outputs

S4.1. Figure S3: GPT-4 Prompt for UC-2 Deidentified Clinical Note Dataset

```
System Role: Pretend you are an oncologist reviewing a clinical note to determine whether CAR-T therapy was discussed for a patient with multiple myeloma and define eligibility.

Eligibility Criteria for CAR-T Therapy in Multiple Myeloma:
A patient may be eligible for CAR-T therapy if they meet all the following conditions:
Confirmed diagnosis of multiple myeloma. Disease has not responded or has relapsed after four or more prior lines of treatment.
Prior therapies must have included:
A proteasome inhibitor, an immunomodulatory drug, and a anti-CD38 monoclonal antibody.

User Prompt: Patient's note ```{patient's clinical note}```.
Please accomplish the following tasks: (1) Was CAR-T discussed? [yes/no], (2) Is the patient eligible for CAR-T? [yes/no/unclear], (3) Provide the rationale for the eligibility determination, and (4) Classify the eligibility rationale for CAR-T discussions into predefined groups: ("Ineligible due to disease criteria", "Ineligible due to frailty or comorbidities", "Ineligible due to social determinants of health", "Ineligible due to not enough prior lines of therapy", "Eligible now or potentially eligible in future", "Not enough information to determine eligibility", and "Other"). \
```

S4.2. Table S6: Eligibility for UC-2 patients who discussed CAR-T therapy

Is the patient eligible for CAR-T?	Count
Yes	6
No	4
Unclear	8

S4.3. Table S7: Eligibility for UC-2 patients who did not discuss CAR-T therapy

Is the patient eligible for CAR-T?	Count
Yes	4
No	136
Unclear	104

S4.4. Table S8: Selected Annotated GPT-4 Prompt Outputs for UC-2 Deidentified Clinical Note Dataset

Patient	A_P	output	TZ Note Annotation Comments
1	In *****, *****. ***** ***** ***** is a ***** y.o. female diagnosed May 2021 with IgA kappa monoclonal " gammopathy, with May 2022 rise in M protein to to 2.4g/dL and BMBX around that time revealing 80% PCs meeting ***** CRAB criteria for multiple myeloma. - BMBX with 80% PCs (UCSF review), standard risk by FISH - PETCT August 2022 negative - Hgb, Cr, Ca nml #) ZOOM ISSUES - must have EVENING VV appointment - 02/02/23 - today the pts son ***** - will help with zoom using his phone - he confirmed in person. - lives in ***** ***** cannot come in person unless needed as she has 3 young kids and no driver LOT#1) = Daratumumab/RVd started September 2022 - ***** May 2023 - RESPONSE = sCR - BMBx and multiple myeloma [MM] labs August 2023 (***** ID failed) - start maintenance daratumumab / lenalidomide August 2023, reviewed with Dr ***** - ***** vaccine August 2023 - encouraged obtaining - bmt vaccines start November 2023 (at 6m, 9m, 12m, 18m)- ?ucsf vs locally MULTIPLE MYELOMA [MM] #) TREATMENT - BMBx: getting 08/21/23 with Dr *****, with multiple myeloma [MM]-FISH - LABS: multiple myeloma [MM] labs q4 weeks with Dr ***** - BONE: bone imaging with PETCT q12 months - maintenance daratumumab / lenalidomide start at *****: around 08/15/23, messaged with Dr ***** - daratumumab q2 weeks x 4 months > then q4 weeks continuous - lenalidomide 10mg 21/28 with vte prophylaxis [ppx] - rtc ucsf 6m VV - requested - rtc November 2023 for 6m set of vaccines - requested #) VTE PPx - continue while on imid #) IMMUNOCOMPROMISED - vzv prophylaxis [ppx] to continue - pjp prophylaxis [ppx] ***** after HCT - ***** vaccine, stay up to date - annual flu vaccine #) BMT VACCINES HBC-Hematology Auto/***** Transplant Vaccination Schedule: - ***** | start @3-months after Auto-HCT -@*****-*****: *****, dT, actHIB, IPV, *****-B >> start November 2023 > will do at ***** (as per August 2023 conversation) (but she will also ask Dr *****) - @*****-*****: *****, dT, actHIB, IPV, ***** >> February 2024 (requested August 2023) -@*****-*****: *****, Boostrix " (Tdap), actHIB, IPV, *****-B -@*****-*****: ***** -@*****: flu vaccine - with primary team Last updated: April 2023-SA #) BONE - after HCT, ***** x1 year - exercise - continue vitamin d and calcium ::OTHER MEDICAL) #) HTN - on home meds, was on amlodipine #) DM #) Iron deficiency anemia with low iron stores in ***** - recommend IV iron #) Social - lives close to ***** *****. Has 3 children, young. Works. No tobacco/EtOH. She is on disability. Married. - will need aggressive social support through the stem cell collection process +/- transplant (if she chooses to proceed). - low health care literacy ***** *****, MD Hematology, Blood & Marrow Transplant, and Cellular Therapy (HBC) *****.*****@*****.***** | office: ***** ***** ***** 08/17/2023 Patient ***** *****: ***** *****, MD as PCP - General (Hematology and Oncology) ***** ***** *****, MD (Hematology) Problem: diagnosis that requiring intensive monitoring Risk: high; requires regular and intensive monitoring for potential major toxicities. Data review/interpretation: reviewed and/or ordered 3+ tests and/or documents and interpreted the tests documented above. My location is in a UCSF clinical facility. "	{ "Was CART Discussed?": "no", "Is the patient eligible for CART?": "no", "Rationale for Eligibility choice": "The patient has not undergone four or more prior lines of treatment, which is a requirement for CAR-T cell therapy eligibility." }	Accurate

2	1. Multiple Myeloma: Impression: established - stable - on therapy - requiring disease monitoring This patient is on therapy and at high-risk for side effects and progression and requiring intensive monitoring. The patient's therapy is lenalidomide - tolerating it well. We reviewed the labs: *****, SPEP, *****, CBC => *****(-), SPEP(-), KLC 19.1, LLC 13.5. MRI August 2023 - old/dense compression fracture. No signs of progression. We discussed that he should continue lenalidomide. No dose adjustments The patient has a high complexity problem: multiple myeloma, which is an incurable malignancy and the patient is receiving active therapy, which have side effects and poses a threat to life and bodily function. Regarding data complexity, I have reviewed the following tests => SPEP, *****, *****, CBC and CMP. Medical Decision Making/ Plan: - Continue on Lenalidomide therapy - Continue to follow myeloma protein levels every 4-6 weeks - Follow-up in 3-4 months 2. Immunocompromised state/Infectious Disease Concerns: Impression: established - immunocompromised from disease/treatment: The patient is immunocompromised and is at high-risk from infection due to therapy. Needs to be up-to-date on Covid vaccine. Has been sheltering from exposure. No exposures. Requiring close follow-up for symptoms. At risk for VZV reactivation and is on ACV. No current signs of infection. ***** call with new signs of infection Plan: - Continue prophylaxis - yearly flu vaccine - ***** from ***** exposure - can get booster this ***** - The patient will call with any symptoms or signs of infection 3. Depression/Psychologic/Social Stressors: Impression: established - stable The patient has cancer and has situation depression and is at risk for anxiety. Needs close follow-up Currently, the attitude is good. There are no signs of anxiety or active depression. Mood appropriate, understands plan Plan: No additional medications needed 4. Anemia: Impression: established - stable The blood counts are good, Hgb 12.4. Next CBC in 4-6 weeks. Plan: No transfusions or cytokines needed today. 5. Neutropenia/Thrombocytopenia: Impression: established - stable The treatment has toxicity on the bone marrow - no current signs of suppression Plan: No cytokines or transfusions 6. *****/ Electrolyte Replacement: Impression: established - stable Taking ample fluids and avoiding nephrotoxins. Lytes & Cr Okay, appears euvolemic. Appetite good and weight unchanged. No new medications needed. Plan: Continue adequate fluid intake & balanced, nutritional diet - Continue as much exercise as possible. - No additional supplementation needed. 7. Osteopenia: Impression: established - stable: The patient is at risk for fracture due to MM. We recommend Calcium and Vit D replacement. We also recommend an aggressive exercise program. Plan: - No Biphosphonates 8. Secondary Cancer Risk: impression: established - NEEDS close follow-up The patient has high-risk of secondary malignancies. Needs close follow-up and routine cancer screening. Plan: - Derm follow-up yearly - Colonoscopy every 5 years - Screening per PCP	{ "Was CART Discussed?": "no", "Is the patient eligible for CART?": "unclear", "Rationale for Eligibility choice": "The clinical note does not provide enough information about the patient's previous treatments to determine eligibility for CART therapy. The patient is currently on lenalidomide therapy, but it is unclear if they have previously received a proteasome inhibitor, an immunomodulatory drug, and an anti-CD 38 monoclonal antibody, which are required for CART therapy eligibility." }	Accurate

3	# Multiple myeloma. The patient was initially diagnosed in 2016 with extensive plasmacytoma but minimal bone marrow involvement. Received 7 cycles of Velcade Cytoxan and dexamethasone followed by out of the stem cell transplant on 04/28/2016 with essentially CR. The patient did not received maintenance therapy but was followed closely. Bone marrow biopsy in July 2020 showed 5% plasma cells consistent with residual disease. At the time lambda light chain was 86.9. The patient started on VRD in November 2020. PET scan in February 2021 did show some active disease but also mixed response. PET scan looks to be improved<br>August 2021 ***** with variable PC infiltration 5-50%, lambda has increased but pt has been off therapy for several months Now s/p 2nd autologous stem cell transplant with melphalan 200 mg/m¬≤ (D1=01/24/22). He essentially had over 4 years of remission without any maintenance therapy thus we though he could do well with the second transplant and lenalidomide maintenance therapy. Plan was supposed to follow-up with Dr. ***** but lost-to-follow-up. Recommended that he follow-up with Dr. *****. Started on rev maintenance 10 mg D1-21q28 days. Progressed April 2023 with worsening AKI and found to have progressive disease s/p urgent plasmapheresis (April 17, April 20-April 22) followed by chemo with *****-*****/*****/D in April 2023 (unclear how many cycles completed). April 2023 ***** 90% L-PCs ***** 3404 04/17/23 > 1666 on 04/22/23 > 1258 on 04/27/23 > 1413 on 05/26/23 > 1725 on 05/01/23 > 1879 on 06/22/23 Switched to Carfilzomib, pomalidomide (dose reduced), dexamethasone (?) - 09/19/23 labs: M-spike 0.1, ***** ***** light chain, LLC 1928. - 09/23/23 24 hour urine collection: 74% M-protein with lambda light chain. Started ***** 10/21/23 - > ***** 6243 -> nadir 851 (but while on PLEX) Subsequently developed PD by 11/10/23 - continued for another month -> without improvement 10/29/23 H/o grade 1 CRS with ***** s/p ***** x 1. Resolved. 12/22/23: ***** ***** 12/23/23: Started on VD-PACE x 1 (with dose reductions for kidney function) - tolerated fairly well 01/26/24: ***** 4.7, ***** 619.4 - most c/w with at least partial response 02/07/24 C1D1 Teclistamab 02/13/24: K 5.1, L 1467, K/L <0.01, M protein 0.2, ***** ***** 02/18/24 ***** 3, L 2362, K/L <0.01, IgG 645 03/09/24: Pomalidomide added to Teclistamab, ***** *****.9, IgG 584, 04/11/24: KLC 4.1 ***** *****, ***** <0.01 Admitted 04/24/24-05/22/24 for ***** c/b by multifocal PNA, blood cultures Positive for corynebacterium, atrial fibrillation Discussed options of: - Trying Selinexor/Velcade/***** - Continuing<br>with alkylator based ***** - given that he has been responsive to high-dose therapy - although with infectious complications. We will proceed with option 1- ***** Multiple myeloma not having achieved remission (CMS code): Discussed initiating treatment with Selinexor, a chemotherapy pill taken once a week, in conjunction with Velcade shots to be managed by Dr. *****. Treatment aims to control the myeloma, which has not yet achieved remission. Selinexor can cause significant nausea, so a comprehensive anti-nausea regimen will be implemented, including dexamethasone, Zofran, and rolapitant - ***** ***** to contact patient or patient's daughter, *****/*****, to arrange delivery of Selinexor and anti-nausea medications. - Patient will take dexamethasone on the day of chemotherapy, Zofran for three days post-chemotherapy, and ***** i on the first day and then every two weeks. - Monitor light chain levels weekly through labs with Dr. *****. - Patient to follow up with Dr. ***** on the January and 5th for labs and to ideally begin BTZ - Patient to contact the cardiologist for follow-up, as referred by the hospital. - Schedule another check-in in about two weeks to assess tolerance and effectiveness of the treatment. Plan: ***** be to attempt selinexor-bortezomib-dexamethasone based on ***** trial. -***** dose reduce selinexor to 60 mg weekly, velcade 1.3 mg SC weekly 3 weeks on and 1 week off, and dex 20 mg weekly OK. - Velcade to be arranged by Dr. ***** locally. - Monitor myeloma marker Q week by Dr. ***** # Immunocompromised # Multifocal pneumonia, likely aspiration # Blood cultures with corynebacterium # Hx of Influenza A PNA # Hx of COVID pneumonia # Hypogammaglobulinemia # Acute Hypoxemic respiratory failure At risk for infections given underlying malignancy and chemotherapy. Recently treated for COVID and FLU pneumonia. He reports to ongoing chronic cough<br>since last admit and some shortness of breath with exertion. No new fevers, chills. Hospital course complicated by recent tachycardia, worsening hypotension, and worsening hypoxia. He initially had viral infection with covid and H3 flu A diagnosed on April 11 for which he received remdesivir and tamiflu (April 12-April 16). CT obtained on April 26 showed no PE or evidence of pneumonia. CXR obtain on May 06 for rising O2 requirement showed decreased right pleural effusion and stable interstitial opacities. CT chest obtained on May 07 was consistent with a multifocal pneumonia. Infectious work-up obtained May 08 showed negative legionella urine antigen, negative coccidiodes antibody, negative toxoplasma ab IgG and IgM, negative galactomannan antigen, negative beta-d-glucan, and negative MRSA swab. Blood cultures from May 07 ultimately returned positive for corynebacterium striatum group which in discussion with ID likely originated with the patient's multifocal pneumonia. TTE was obtained and showed no signs of infection. Patient continues to have O2 requirement of 1-2L which is likely related to pneumonia and less likely volume overload. Bumex currently on hold iso soft blood pressures. Per SLP eval, there were no obvious signs of aspiration; pt continued on regular diet with aspiration precautions. Plan: - Infectious disease consulted, appreciate recs - Continue Zosyn (May 07-) and Vancomycin (May 08-), x 14 day course - complete June 01	{<br><br>"Was CART Discussed?": "no",<br><br>"Is the patient eligible for CART?": "yes",<br><br>"Rationale for Eligibility choice": "The patient has a confirmed diagnosis of multiple myeloma and has undergone multiple lines of treatment including a proteasome inhibitor, an immunomodulatory drug, and an anti-CD 38 monoclonal antibody. The disease has not responded or has relapsed after these treatments, making the patient eligible for CAR-T cell therapy."<br><br>}	Accurate

	- ***** PICC line removed May 10 - will plan for vancomycin lock for tunneled HD line as it seems more likely that this infection originated from ***** as opposed to line related. [x] ***** obtain repeat Bcx from ***** cath and periphery on May 24 (After HD, dialysis aware); if recurrent bacteremia will likely need to exchange tunneled dialysis catheter. - > negative - TTE w/o findings c/f infection - continue incentive spirometer - albuterol q6hrs while awake and prn - Ppx acyclovir 200mg at bedtime - Dc'd iso for COVID on May 03 # New atrial fibrillation with RVR No prior documented hx of this. Likely to be in the setting of sepsis/PNA. S/p IV amiodarone and now on PO to complete load. He will start maintenance amiodarone on May 14 and follow-up with cardiology on discharge. He will also need a ***** on discharge. ***** 4 however unable to administer AD due to low plt. -PO amiodarone load finished May 13, started 200mg maintenance on May 14 - Recommend ***** and cardiology follow-up on discharge [ ] advised him to call cards clinic to schedule follow-up. # Immunodeficiency - Continue acyclovir. - Continue Mepron for PCP ***** #. Hypogammaglobulinemia - Give IVIG for IgG < 400. #. Anemia, thrombocytopenia - continue to monitor #. Bone disease. He was on ***** - on hold after transplant recovery. #. History of pulmonary embolus, 2015 #. New DVT, 09/19/23, w/ likely acute PE - PE diagnosed in August 2015 . He was previously on Coumadin. ASA ppx while on revlimid, d/c upon completion of Revlimid. Restarted ASA with Pomalidomide. - 09/19/23 RLE Dopplers: Duplicated right femoral vein with occlusive thrombus along one of the duplicated veins extending to the popliteal, peroneal and posterior tibial veins - 09/21/23 V/Q scan: Single matched ventilation and perfusion defect on the left. Based on ***** criteria, intermediate probability for pulmonary embolism - Heparin gtt (September 19-September 22) transitioned to apixaban (September 22) Plan: - Continue apixaban 2.5 mg BID. Reordered February 18. #. At risk for TLS - Continue allopurinol. # AKI on CKD suspect November 06 light chain nephropathy, now on HD # Hypocalcemia and hypophosphatemia Prior Cr of 1.12 (December 2022) c/b severe AKI April 2023 with Cr peak 4 In the setting of progressive MM and light chain nephropathy. Improved with fluids. - September 19 LDH and uric acid within normal limits. - September 20 renal US suggestive of medical renal dz, no hydronephrosis or nephrolithiasis - September 21 no retention on bladder scan, urine studies consistent with intrarenal etiology Plan: - CTM, avoid nephrotoxins - HD on Tue/*****/Sat. - Followed by local nephrology, Dr. ***** (office: *****-*****-*****, cell: *****-*****-*****). - de-escalate to 2mg bumex daily on non-HD days - ***** to 1600 mg TID ***** due to elevated phos - plan for aranesp with HD - Has a chest port. Scheduled to get AV fistula on May 06. Uncertain if he will be stable enough for the procedure. - Spoke with Dr. ***** and in agreement to reschedule the fistula. Would like to know when the patient is going to get more treatment after VD-PACE so that he can coordinate fistula placement. #. Abnormal uptake on prostate. # BPH. Dr. ***** previously discussed with local oncologist whether he has had PSA checked and had this not worked up yet. Repeat PET without uptake but enlarged prostate #. Cancer-related pain Mild-moderate low back pain reported today; improving since February 13. Of note, reported MVA accident on February 13 after being discharged from hospital. -continue oxycodone prn - CT Thoracic/Lumbar spine non con ordered February 19. Pt called with number to call and schedule. - Missed scheduled CT on February 25. Rescheduled for February 26 but patient cancelled. ***** order CT to be completed locally. ***** *****, MD Assistant Clinical Professor UCSF - Hematology/Bone Marrow Transplantation ***** cell - *****-*****-*****		
